# Beyond the blank slate: routes to learning new coordination patterns depend on the intrinsic dynamics of the learner—experimental evidence and theoretical model

**DOI:** 10.3389/fnhum.2012.00222

**Published:** 2012-08-03

**Authors:** Viviane Kostrubiec, Pier-Giorgio Zanone, Armin Fuchs, J. A. Scott Kelso

**Affiliations:** ^1^PRISSMH, F2SMH Université de ToulouseUPS, France; ^2^Center for Complex Systems and Brain Sciences, Florida Atlantic University, Boca RatonFL, USA; ^3^Intelligent Systems Research Centre, University of Ulster, Magee CampusDerry, UK

**Keywords:** coordination dynamics, stability, bifurcation, dynamical systems, relative phase, perceptual-motor coordination, modeling

## Abstract

Using an approach that combines experimental studies of bimanual movements to visual stimuli and theoretical modeling, the present paper develops a dynamical account of sensorimotor learning, that is, how new skills are acquired and old ones modified. A significant aspect of our approach is the focus on the individual learner as the basic unit of analysis, in particular the quantification of predispositions and capabilities that the individual learner brings to the learning environment. Such predispositions constitute the learner's behavioral repertoire, captured here theoretically as a dynamical landscape (“intrinsic dynamics”). The learning process is demonstrated to not only lead to a relatively permanent improvement of performance in the required task—the usual outcome—but also to alter the individual's entire repertoire. Changes in the dynamical landscape due to learning are shown to result from two basic mechanisms or “routes”: bifurcation and shift. Which mechanism is selected depends the initial individual repertoire before new learning begins. Both bifurcation and shift mechanisms are accommodated by a dynamical model, a relatively straightforward development of the well-established HKB model of movement coordination. Model simulations show that although environmental or task demands may be met equally well using either mechanism, the bifurcation route results in greater stabilization of the to-be-learned behavior. Thus, stability not (or not only) error is demonstrated to be the basis of selection, both of a new pattern of behavior and the path (smooth shift versus abrupt qualitative change) that learning takes. In line with these results, recent neurophysiological evidence indicates that stability is a relevant feature around which brain activity is organized while an individual performs a coordination task. Finally, we explore the consequences of the dynamical approach to learning for theories of biological change.

## Introduction

Recent evidence demonstrating that resting brain activity (i.e., in the absence of external stimulation) consumes as much as 60–80% of the total brain energy devoted to neural processing (Raichle and Gusnard, 2005) has led to renewed interest in the role of spontaneous activities in the behavioral and brain sciences (Smallwood and Schooler, 2006). Neural processes and their manifestations, mentation, and behavior, are said to be spontaneous if they are endogenously generated and progress on their own, without any induction or prescription from the environment. In the context of learning, spontaneous activity refers to the learner's predispositions or tendencies that provide the background upon which to develop and shape task- or goal-dependent activities. In the present article, we shall apprehend spontaneous activity at the neural and behavioral levels in the language of coordination dynamics, a theoretical and empirical framework aimed at understanding the (directed) self-organization of coordinated patterns of behavior in complex systems (see Kelso, 1995; Tschacher and Dauwalder, 2003; Kelso and Engstrøm, 2006; Kelso, 2009; for reviews). In coordination dynamics spontaneous activity is captured by the concept of *intrinsic dynamics*: any changes due to learning are therefore seen in the light of pattern stability, instability, fluctuations, and so forth. In the following, we shall address the issue of behavioral change at several levels of description through the window of human sensorimotor coordination and skill learning.

From the outset of experimental psychology to the present day, experimenters have done their outmost to reduce the impact of spontaneous activities on the processes of learning, recall, forgetting, and transfer (Ebbinghaus, 1885; Thorndike, 1911). Instead, attention has largely been confined to behaviors built up by experience, thereby avoiding *ex-post facto* any explanation that summoned spontaneous activity as a key player in learning (Thorndike, 1911; Bitterman, 1975). As a consequence, seldom has learning theory considered spontaneous activities to be the initial backdrop upon which learning operates. On the one hand, spontaneous behaviors were first thought of as biologically prepared, stereotypical responses, providing at best a “helping hand” to learning (Thorndike, 1911). On the other hand, they were equated with accidental, purposeless behaviors the function of which was to build up a “raw” behavioral repertoire of initial responses, later to be shaped by learning processes (Estes, 1959; Skinner, 1990; Elsner and Hommel, 2001). Although the learner's spontaneous behavioral repertoire was acknowledged to play a pivotal role in learning failures (Breland and Breland, 1961; Seligman, 1970; Garcia et al., 1974; Shettleworth, 1978; Timberlake and Lucas, 1989; see Johnson, 1981; Timberlake, 1990, for reviews), a specific analysis of such effects has proven difficult in the extreme. Experimenters lacked strategies, conceptual tools and operational measures that would enable them to make deeper experimental investigations of the role of initially present patterns of behavior in individual learners. In the present contribution, which meshes experimental evidence and theoretical modeling, the pre-existing repertoire of spontaneous behaviors in the individual learner is captured formally by the notion of intrinsic dynamics, i.e., in terms of patterns in the repertoire and their stability.

Scientific endeavor is thought to aim at discovering simple and universal laws that allow for generalization, prediction, and simulation. Such is the case for learning. Laws of learning have long been defined by averaging performance across multiple participants, as exemplified by typical power laws (Newell and Rosenbloom, 1981; Newell, 1991; Newell et al., 2006, for review). Under this umbrella, any account of pre-learning spontaneous behavior in a given individual is relegated to the relatively uninteresting or unmanageable presence of inter-individual differences and tends to hide general laws of learning. In the present paper, by means of experimental studies motivated by the concepts, methods and tools of coordination dynamics, we aim to show that simple and universal laws of learning and associated processes of attention and memory are formed on the basis of spontaneous behaviors that are specific to individuals rather than despite them.

Performance in learning and memory studies is typically assessed by *accuracy*, that is, by the mismatch between what is required (e.g., a task goal) and what is produced. Here, accuracy is measured by the absolute distance between the required pattern and the average of the produced patterns in a trial, that is, by the absolute value of the constant error. Yet, when behaviors are generated on their own, no required behaviors and thus no accuracy measures can exist. Inspired by theories of self-organization and spontaneous pattern formation in non-equilibrium physical and chemical systems (Nicolis and Prigogine, 1977; Haken, 1983; Bushev, 1994; see also Kugler et al., 1980), coordination dynamics aims to solve this problem by casting a novel look on a so-far misunderstood and neglected feature of performance: the *stability* of the produced behavioral patterns. One of the main findings of the present work is that the paths that different people take to learning a new skill—whether the skill is acquired suddenly or gradually, how much attention is devoted to learning a new skill and people's ability to recall that skill—are a function not only of improvements in accuracy of the behavioral patterns produced over the course of practice, but also, and above all, of changes in pattern stability.

We should clarify what we mean by stability. Theoretically, stability pertains to resistance to change, captured by the time it takes for a system to relax back to its initial state after a perturbation has driven it away (Schöner et al., 1986). Behaviorally, the more stable is the pattern produced, the shorter the time to return to its initial state. Operationally, stability is often gauged by variability, typically assessed by standard deviation (SD), indicative of fluctuations in the current state over time (Schöner and Kelso, 1988a, for review). Over all ideally possible patterns, only those which are stable enough can be spontaneously generated and maintained in the face of naturally occurring perturbations, thereby constituting an individual's behavioral repertoire. This is why such spontaneous patterns are often called *preferred*. High stability defines spontaneous patterns which, in a given context, may represent biases and predispositions when the learner is faced with acquiring a new behavior.

At the core of self-organizing processes lies *loss of stability*: a behavioral pattern may destabilize to such an extent that a sudden, abrupt switching occurs to another form of behavior due to an increase in the constraints imposed on behavior (Kelso et al., 1987). In physics, such switching is called a *nonequilibrium phase transition* (Haken, 1983) and may take the mathematical form of a *bifurcation* (e.g., Guckenheimer and Holmes, 1983; Strogatz, 1994), which is brought about by changes in so-called *control parameters*. Of significance to the issue of learning is that transitions are quantifiable manifestations of a qualitative re-organization of the spontaneous activities that constitute the current behavioral repertoire (viz. intrinsic dynamics). Far from being mere noise or a measure of uncertainty (Trommershäuser et al., 2003; Körding and Wolpert, 2004), increase in variability precedes bifurcation and is thus a meaningful feature, informing on whether and how an organism re-organizes its spontaneous activities (Schöner and Kelso, 1988a; Slifkin and Newell, 1998; Scheffer et al., 2009).

Perhaps the simplest instance of self-organization in a biological system comes from experiments on the coordinated action of cyclical movements of the limbs. When participants are asked to move their right and left index fingers back and forth in the horizontal plane, they typically exhibit only two spontaneously stable bimanual coordination patterns (Kelso, 1984): The fingers move either in opposite directions, resulting in an in-phase coordination pattern, or in the same direction, hence an anti-phase pattern. Relative phase, a measure of the time lag between the moving fingers (e.g., Pikovsky et al., 2001), amounts to 0° for the in-phase and to 180° for the anti-phase coordination pattern. Over a typical trial, the 0° relative phase pattern turns out to be consistently more stable than 180°: its variability, assessed by the standard deviation of relative phase, is lower, and the time to return following a brief perturbation faster (Kelso et al., 1986, 1987). When the 180° pattern must be produced at higher movement frequencies, its variability increases systematically, an instance of predicted critical enhanced fluctuations prior to a phase transition (Kelso et al., 1986). At some critical frequency, a sudden switching to the more stable 0° coordination pattern occurs, as only the 0° pattern can be sustained at higher movement frequencies (Kelso, 1984). In-phase and anti-phase patterns prove to be a universal feature of motor coordination. When subjects are requested to perform typical activities they would do normally, such as walking, doing jigsaw puzzles, preparing food and having lunch, in-phase and anti-phase appears as the most often produced pattern between the limbs (Howard et al., 2009). They also arise as a stable relationship in sport activities, such as cross-country skiing (Cignetti et al., 2009), gymnastics (Delignières et al., 1998) and the volley-ball serve (Temprado et al., 1997). It is important to recognize that these spontaneously stable patterns do not appear to be the result of practice or special learning procedures. Rather, in-phase and anti-phase patterns may be said to constitute the individual's *intrinsic dynamics* prior to any practice. Of course, this hypothesis must be examined independently when it comes to learning a new pattern of coordination, that is, one that does not belong to the individual learner's pre-existing behavioral repertoire.

In coordination dynamics, the modeling strategy is to map observed, reproducible, stable patterns of behavior onto *attractors* of a dynamical system, that is, stable solutions of a formal equation of motion, which determine how the states of the system evolve in time. In the case of bimanual coordination, such dynamics stipulates the rate of change of an explicit time-evolving variable, the relative phase (ϕ) between the movements of the two fingers. For example, if the two coordinated patterns, in-phase and anti-phase, are possible at the same frequency, the dynamics of ϕ is *bistable*, displaying two attractors at 0° and 180° (Haken et al., 1985). Of course other possibilities may exist as well. For example, an individual learner may enter the learning situation with three or more stable coordination patterns and hence exhibit *tri- or multistable* dynamics. The key point is that when it comes to understanding the coordination dynamics of learning, a major task is to empirically identify an individual learner's current behavioral repertoire (viz. intrinsic dynamics) prior to learning (Schöner and Kelso, 1988b; Kelso, 1995). Once this step is achieved, subgroups of learners that share a common initial repertoire may be formed, thereby opening up the possibility of identifying general mechanisms and principles governing how people change during the learning process (Zanone and Kelso, 1992a, 1997).

## Results on learning, attention, recall and transfer

To address changes due to learning it is necessary to devise an operational means to probe the individual learner's repertoire before, during and after the learning process. Before an individual even begins learning, the basic idea is to confront him or her with a wide range of task requirements not previously encountered (Yamanishi et al., 1980; Tuller and Kelso, 1989). In our research paradigm, several visually specified relative phase patterns between the moving limbs are presented in sequence, a procedure referred to as a “scanning probe” (Zanone and Kelso, 1992a, 1997; Kelso and Zanone, 2002). Set in a dim-lighted room, participants are required to produce coordination patterns by flexing and extending their index fingers back and forth on the horizontal plane (e.g., Zanone and Kelso, 1992a) or rotating their wrists in the frontal plane (e.g., Kostrubiec et al., 2006). Notice only kinesthetic or proprioceptive information is available for movement; this is not a tapping task where haptic information is known to play a stabilizing role (e.g., Kelso et al., 2001). Two light emitting diodes (LEDs) specify a range of required relative phase patterns in the 0–180° interval, by steps of 15°. For example, LEDs blink simultaneously to specify 0° and alternate to specify 180° as the required pattern. The participant's task is to synchronize the right and left finger or hand movements with the onset of the right and left LEDs, respectively. No explicit feedback on error is provided during this initial scanning phase of the experiment. The significance of scanning probes is that they reveal the presence of preferred or stable behaviors: were there none prior to learning, all task requirements should be met equally well, all relative phase patterns should occur equally often and be produced with the same (presumably poor) accuracy and/or variability. In contrast, the presence of a pre-existing repertoire should render some patterns required in the scanning probe more frequent, more accurate and/or more stable than others. In the present paper, a composite measure of accuracy and stability, root mean squared error (RMSE), is used to reveal the underlying (coordination) dynamics: The smaller the RMSE the greater a pattern's accuracy and stability, two signature features of attractors. Note that in order to obtain a more precise and meaningful understanding of the underlying dynamic landscapes (viz. individual repertoire), the two measures should be considered separately, as is the case in all our studies.

**Figure 1:**
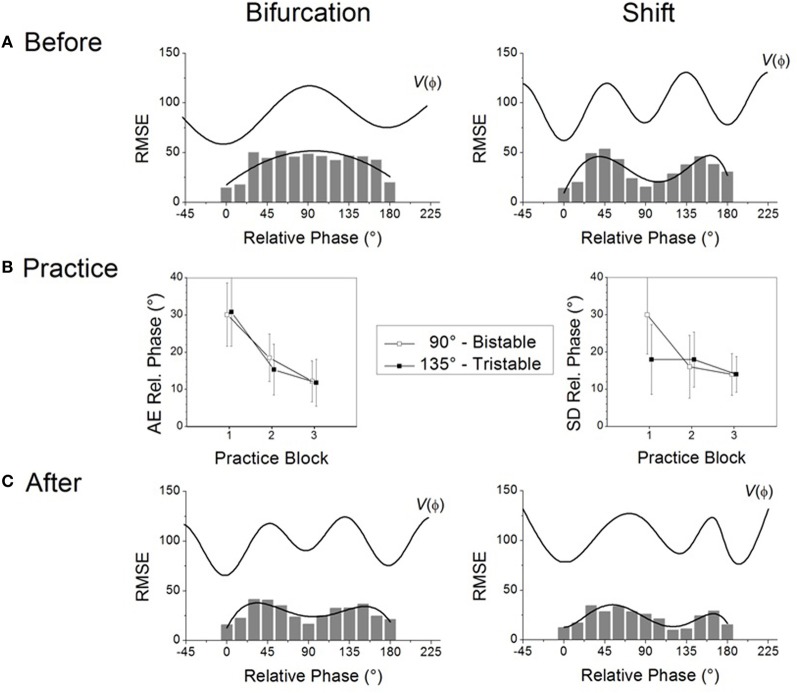
**Two routes to learning**. See text for details. (Data adapted from Kostrubiec et al., 2006).

What do the intrinsic dynamics look like in typical experiments? In a normal adult population, scanning probes give rise to two sets of results before learning (Figure 1A). The most common experimental finding is that even though different relative phases are specified in the scanning, the 0° and 180° patterns are produced most frequently. Data also show that these patterns are more accurate and more stable, as indicated by low RMSE at these values, emphasized by a polynomial fit (Figure 1A left), and that they influence the performance of other relative phases—a manifestation of attraction (Tuller and Kelso, 1989; Zanone and Kelso, 1992a; Hodges and Franks, 2000, 2002; Hodges et al., 2003; Atchy-Dalama et al., 2005; Maslovat et al., 2005; Kostrubiec et al., 2006; Tallet et al., 2008; Zanone et al., 2010). All extant experimental findings lead one to the conclusion that 0° and 180° are candidate attractors of the coordination dynamics before learning. For illustrative purposes, an idealized representation of the corresponding dynamical landscape is given above the RMSE distribution in Figure 1A (left) by a potential curve *V*(ϕ). Later, we will be much more specific about the form of this potential function. For now, we remark that such a landscape is illustrated by two wells, corresponding to minimal RMSE and reflecting two putative attractors located at 0° and 180°. On an individual basis, a pattern was qualified as stable if it could be performed with the absolute value of constant error (AE) and a standard deviation smaller than 20°. The dynamics prior to learning may thus be said to be bistable.

In a smaller number of individuals, the RMSE produced as a function of required relative phase during scanning exhibits three minima in the 0–180° interval (Figure 1A right): two around 0° and 180° and one at 90°. The additional minimum at 90° suggests that this pattern is part of the pre-existing repertoire and that the initial dynamic, illustrated by the potential *V*(ϕ) displayed above the empirical distribution, is therefore *multistable* before learning. It is notable that in every study conducted so far, all individuals regardless of previous experience, say, with music or sports (Verhul and Geuze, 2004; Faugloire et al., 2009) may be classified as bistable or multistable prior to learning. Although a large-scale statistical survey is missing, all accumulated data suggest that in a typical sample of the population, 75% of the participants are bistable and 25% tristable when the required frequency is about 1 Hz.

Knowledge of the preexisting repertoire is instrumental in defining what a new pattern is, and therefore plays a key role in setting what the learning task will be^1^. In coordination dynamics, a new pattern is one that is not already in the learner's repertoire and is therefore bound to compete with the learner's intrinsic dynamics. Theoretically, the new pattern to be learned is a repellor of the dynamics, which can be identified as a “hump” in the potential curve displayed in Figure 1A. Given the two types of repertoire that exist before learning, the relative phase to be learned will be set to 90° for participants who express initially bistable dynamics (0° and 180° only, e.g., left side of Figure 1A) and to 135° for those with multistable dynamics (0°, 180° and 90°, e.g., right side of Figure 1A). Operationally, this means that in both cases the experimenter chooses the pattern to be learned in-between those available in the pre-existing repertoire. This experimental strategy keeps constant the relational, or topological, properties of the to-be-learned pattern within the pre-learning landscape, and ensures competition between the learning task and the behavioral repertoire (viz. intrinsic dynamics) that is present before practice.

During practice, as in the scanning probes, the to-be-learned pattern is specified visually by the LEDs that participants are instructed to match by producing an adequate coordination pattern between the moving fingers. But unlike scanning probes, feedback on the accuracy and variability of the performed pattern is provided after each trial. How do learners handle these new learning tasks? Regardless of whether one is initially bistable or multistable before learning, the mismatch between the produced and the required pattern diminishes, as captured by a reduction in the absolute value of constant error which tends to zero as a function of practice (Figure 1B left). This finding indicates a substantial improvement in the accuracy of the produced pattern. But does such improved accuracy mean that the same underlying mechanism is at work? Not necessarily. Stability is a critical factor. Whereas learning 90° is accompanied by a significant decrease in the standard deviation of relative phase (Figure 1B right, open boxes), no such decrease occurs when learning 135° (Figure 1B right filled boxes). This means that for initially bistable participants practice leads to a noticeable decrease in performance variability, reflecting an increase in the stability of the newly learned pattern. Such is not the case for participants whose pre-learning repertoire is multistable: their variability is already rather low and does not change noticeably over practice trials.

What changes underlie modifications in pattern stability at the level of the coordination dynamics? Some insight may be gained by evaluating the behavioral repertoire after learning by means of scanning probes performed after practice. For those with an initially bistable repertoire (cf. Figure 1A left), the RMSE of the performed relative phase now exhibits three minima: a new one close to 90° appears along with the two pre-learning ones at 0° and 180° (Figure 1C left). The corresponding post-practice potential (see top *V*(ϕ) curve) exhibits a new well located at 90°, reflecting the stabilization of a new attractor at the value required by the learning task. In the case of initially bistable participants, learning has qualitatively reorganized the attractor landscape, the coordination dynamics changing from bistable to multistable. Theoretically, this passage from bistablity to multistability is called a phase transition or bifurcation (Zanone and Kelso, 1992a). We therefore refer to this mechanism of change as the *bifurcation route* to learning (Zanone and Kelso, 1994; Zanone and Kostrubiec, 2004).

In the case of initially multistable learners confronted with the novel task of learning 135° (cf. Figure 1A right), the RMSE as a function of the required relative phase does not change qualitatively (Figure 1C right) compared to the pre-learning repertoire. Three minima are still apparent, but the original minimum at 90° has moved toward 135°. This shift from 90° to 135° is a sign of learning, but the overall attractor landscape is not altered qualitatively, since the number of attractors does not change. Multistability is maintained but only a quantitative alteration is observed in the potential with the relocation of the attractive state at 90° shifting gradually to 135°. Inspired by Schöner (1989), and Schöner et al. (1992), we refer to this mechanism as the *shift route* to learning.

**Figure 2:**
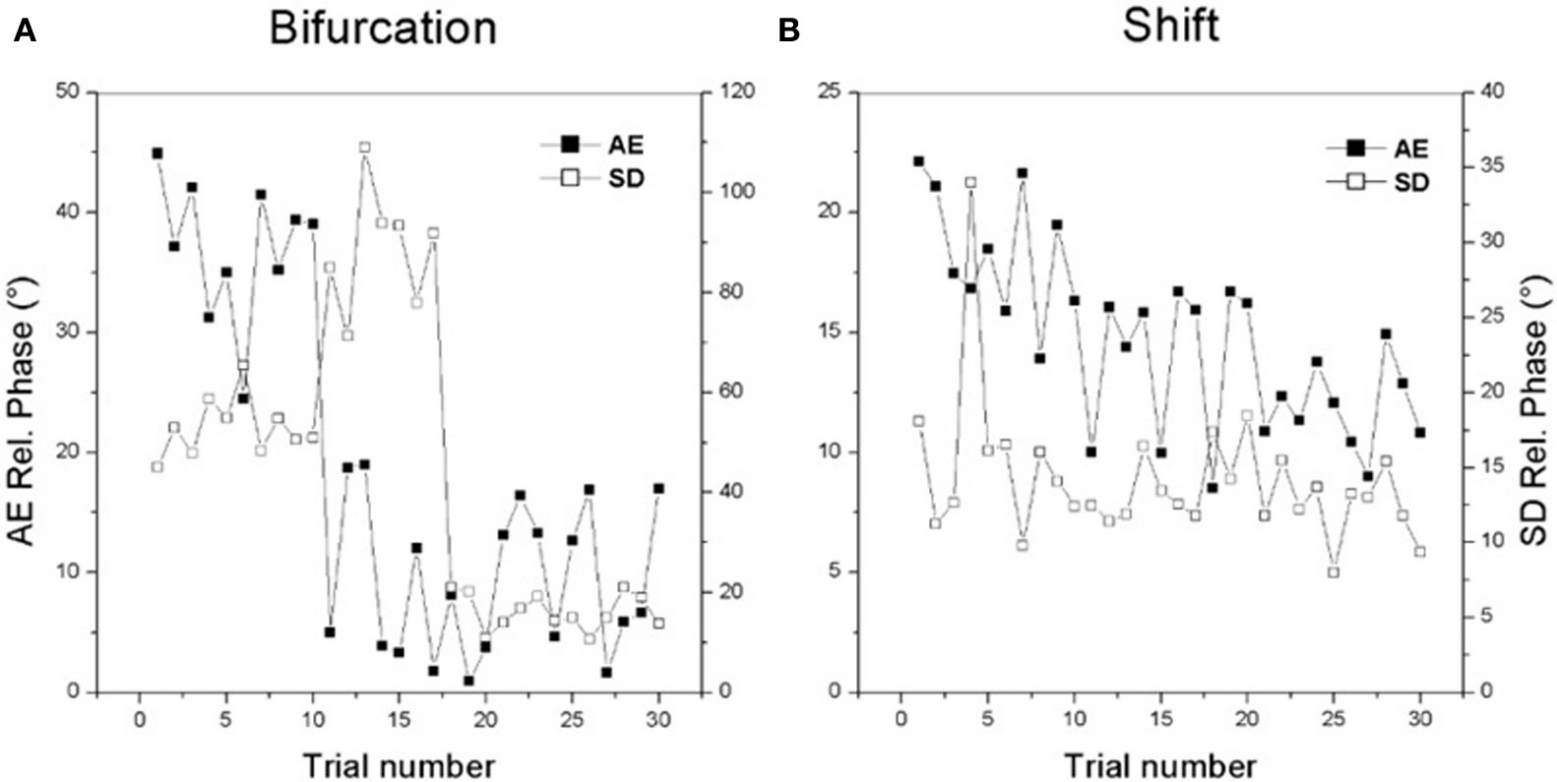
**Changes in individual performance for the two routes to learning**. See text for details. (Data adapted from Kostrubiec et al., 2006).

It is important to note that both the bifurcation and shift routes to learning can be readily seen on a trial by trial timescale as well. Figure 2 shows examples of individual learning. In Figure 2A an initially bistable learner displays the bifurcation route: a sudden reduction in the absolute value of constant error (black boxes) is accompanied by a jump in variability (open boxes) followed by reduction of both quantities. In Figure 2B, an initially multistable learner displays a gradual improvement in performance accuracy (black boxes) over trials with only modest changes in variability (open boxes).

Before accepting the proposal that two different dynamical mechanisms underlie learning, we need to consider their impact on other “higher” cognitive functions typically associated with learning, such as memory and attention. Further experiments (Kostrubiec et al., 2006) have demonstrated that initially bistable participants who improved both the stability and accuracy of the to-be-learned 90° pattern were able to recall the newly created pattern 1 month after the final practice session. In contrast, initially multistable participants, who improved only the accuracy of the to-be-learned 135° pattern by shifting the initially stable 90° pattern toward a 135° value, failed to recall the practiced pattern over the same 1-month interval. Further evidence for different learning mechanisms was obtained by assessing the attentional demands associated with learning using a classic dual-task procedure (Zanone et al., 2010). For initially bistable participants who took the route by bifurcation, attentional demands, measured by discrete reaction time (RT) probes, diminished significantly as a novel behavior is acquired. For initially multistable participants who took the route by shift, attentional cost and variability were low over the whole practice period. On the whole, qualitative, attention-demanding alterations in the behavioral repertoire following the creation of a novel pattern is a persistent effect, whereas the mere shift of an extant pattern toward the required value entails a far shorter-lived, less attention-demanding adjustment. It is important to note that when initially bistable participants learn either a 90° or 135° pattern, they always display the typical hallmarks of bifurcation. Indeed, 135° learning induces first an unsolicited stabilization of the 90° pattern and only then shifts toward the required 135° value (Tallet et al., 2008). Apparently, in the route by bifurcation for learning any novel task is a necessary consequence of a bistable repertoire: only in a second phase are other specific relative phase values eventually reached following a shift route.

To sum up, what we have been able to show experimentally is that changes in overt behavior with practice follow from two radically different mechanisms depending on the initial repertoire of the learner. An initially bistable repertoire follows the bifurcation route to learning, whereas an initially multistable repertoire undergoes a gradual shift. The data also convey another deeper lesson about cognitive processes: sensorimotor learning, memory, and attention, which, to date, have been studied in almost total isolation from each other, may be coherently put back into relationship^2^. By virtue of the dynamic feature of stability, all three processes can now be linked conceptually and empirically: although a similar degree of accuracy and stability may be reached as a result of learning, the bifurcation mechanism invariably yields a larger gain in stability, longer-lasting recall and a higher attentional cost than the shift route.

A last noteworthy result is that learning, whether via bifurcation or via the shift route, does not impinge only on the pattern to be achieved but rather on the whole layout of the coordination dynamics underlying the learner's behavioral repertoire. Previous work (Zanone and Kelso, 1992a, 1997; Kelso and Zanone, 2002; Temprado and Swinnen, 2005) has demonstrated that while a new task is learnt, there is spontaneous transfer of learning to a new unpracticed task. The creation of a stable pattern at 90°, for example, is spontaneously mirrored by that of another stable pattern at −90°, while the shift from 90° to 135° is spontaneously accompanied by a symmetric shift from −90° to −135°. What these findings suggest, along with others showing transfer across different effectors, is that a rule governing transfer of learning may be the preservation of the symmetry of the underlying dynamics (Zanone and Kelso, 1992a,b, 1997; Collier, 1996; Kelso and Zanone, 2002; Zanone and Kostrubiec, 2004).

## Dynamical model of two routes to learning

Here we outline a dynamical model for learning new bimanual coordination patterns that captures all the experimental findings described above. To this end the model for bimanual coordination, originally introduced by Haken et al. (1985), is generalized to model both the bifurcation and shift scenarios of skill learning observed experimentally. Mathematically, in the framework of coordination dynamics, bimanual coordination patterns are described by means of a collective variable, the relative phase, ϕ. The dynamics of ϕ in the original model, known in the literature as HKB is given by a first order differential equation, a relation between the change in relative phase, $\dot{\phi }$, the control parameters *a* and *b*, and ϕ itself (Equation 1):

(1) $$\dot{\phi }=-a\sin\;\phi -2b\sin\;2\phi$$

Equivalently, the dynamics can be captured by a potential function of the form (Equation 2):

(2) $$V\left(\phi \right)=-a\cos\;\phi -b\cos\;2\phi \text{ with }\dot{\phi }=-\frac{dV}{d\phi }$$

In the HKB model stable coordination patterns correspond to minima in the potential landscape or intersections with a negative slope in phase space portraits (diagrams that show $\dot{\phi }$ as a function of ϕ, see Figure 4 bottom) between the function $\dot{\phi }$ and the horizontal axis. Such states are termed stable fixed points or attractors. Correspondingly, maxima in the potential or intersections with a positive slope are unstable fixed points or repellors. Attractors and repellors can be classified by their stability, which is given by the negative of the slope at the intersection in phase space or the curvature at the minima and maxima in the potential. The shape of the function $\dot{\phi }$ and landscape of the potential, and therefore the dynamical behavior of the model system, can be changed (or controlled) by changing the control parameters *a* and *b*.

The present model is based on the assumption that during learning the overall layout of the potential landscape undergoes a change eventually leading to an attractor corresponding to the behavioral pattern to be learned. The form of the potential proposed here consists of two major parts (Equation 3):

(3) $$V\left(\phi \right)=\left\{-\sum_{n=1}^{N}a_{\text{n}}\cos\;n\phi \right\}-\frac{c}{2\pi I_{0}\left(\kappa \right)}\left\{e^{\kappa \;\cos\left(\phi -\psi \right)}+e^{\kappa \;\cos\left(\phi +\psi \right)}\right\}$$

The term inside the first curly brackets, essentially a Fourier expansion with cosine terms only in order to preserve left/right symmetry, represents the potential landscape prior to learning. For initially bistable participants, *a*_1_ and *a*_2_ have finite positive values and all other coefficients vanish, leading to a potential with minima (attractors) at ϕ = 0° and ϕ = 180° as shown in Figure 1A left. We shall refer to the potential shape defined by the coefficients *a*_n_ and the cosine terms as the *skeleton of the potential*. The coefficients *a*_n_ stay constant when no learning or practice occurs but can change during the learning phase.

**Figure 3:**
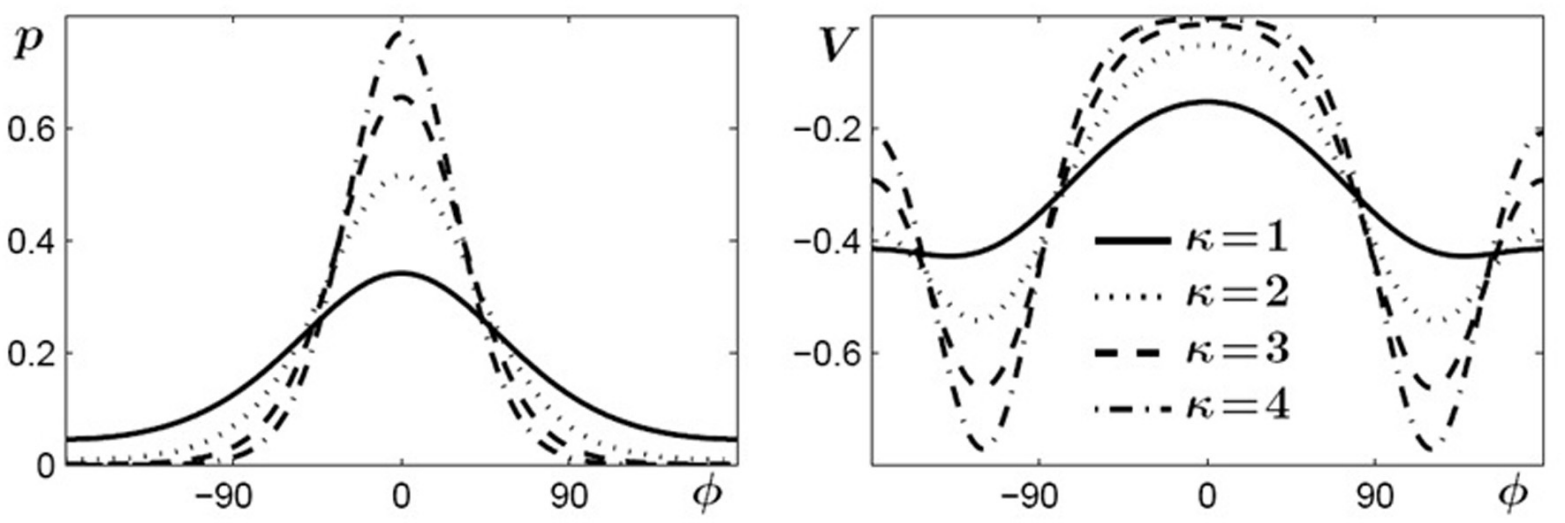
**Von Mises distributions and their use in the potential function (3)**. **Left**: periodic distributions for different values of κ. **Right**: two-well functions used in (3) for different values of κ and for a learned pattern of ψ = ±120°.

The second term in Equation (3) consists of periodic functions, known as von Mises distributions which are essentially Gaussians on a circle, where ψ (or −ψ) represents their mean value and 1/κ is roughly proportional to the variance and describes the width of the distribution. The denominator is a normalizing factor, with *I*_0_(κ) a modified Bessel function of order zero. Two distributions with means at ±ψ are needed because, first, experimental findings show that if a certain relative phase ψ is learned its symmetric partner at −ψ is learned as well (Zanone and Kelso, 1997; Kelso and Zanone, 2002). Second, from a theoretical point of view the potential function must be invariant under a transformation that replaces ψ by −ψ, which corresponds to replacing the left end effector by the right one (handedness not taken into account). In Figure 3 von Mises distributions for different values of κ are shown on the left and examples of the functions used in the potential (Equation 3) for ψ = 120° are shown on the right. These functions, defined by the parameters *c*, κ and ψ, only contribute to the potential as long as learning takes place and shortly thereafter. As a consequence, prior to learning *c* vanishes, increases when learning takes place, reaches a saturation value when the learning phase is sufficiently long, and finally decays on a relatively fast time scale, post-learning.

From the viewpoint of coordination dynamics, to learn to perform bimanual movement with a new relative phase that could not be performed before learning means creating a minimum in the potential landscape (Equation 3) that previously did not exist. Such minima may be realized by a change in the skeleton (the coefficients *a*_n_), by adding other functions like the von Mises distributions mentioned above or both. In order to model the experimental findings of two learning routes depending on whether a given subject enters the learning environment as initially bistable or multistable both ways of changing the landscape are required.

In summary, and to be quite explicit, the model proposed here is based on the following experimental findings:

- Naive learners can be divided into two groups called bistable and multistable. The former can perform in-phase and anti-phase movements only, whereas the latter are able to perform a close to 90° pattern prior to learning;
- When bistable participants learn 90°, the in- and anti-phase patterns remain unaffected. In the potential landscape new minima at ψ = ± 90° arise;
- When multistable participants learn 135°, eventually the 90° pattern is no longer performed. During the learning phase participants perform stable relative phases that move away from 90° toward the required phase. In the potential landscape the minima at ±90° shift smoothly to ±135°;
- After formerly bistable participants learn 90° and are tested at a certain time after learning takes place, they can still produce this phase relation—the minima persist;
- After former multistable participants learn 135° and are tested a certain time after learning takes place, they switch back to a coordination pattern close to 90°; that is, the minima shift towards their original location.

## Simulations: learning of ψ = 90° and ψ = 135°

The temporal dynamics for the relative phase ϕ is given by the negative derivative of the potential *V*(ϕ) with respect to ϕ (Equation 4):

(4) $$\dot{\phi }\;=-\frac{dV\left(\phi \right)}{d\phi }=\;-\sum_{n=1}^{N}n\;a_{\text{n}}\sin\;n\phi -\frac{c}{2\pi I_{0}\left(\kappa \right)}\left\{e^{\kappa \cos\left(\phi -\psi \right)}+e^{\kappa \cos\left(\phi +\psi \right)}\right\}\kappa \sin\;\phi$$

As already mentioned, learning takes place through a change of the potential landscape, that is, a change in the skeleton along with a “force” realized by the von Mises functions, to create minima at the required relative phases. In the model, the skeleton is described by the coefficients *a*_n_, the effects of the von Mises distributions by their amplitude *c*, their width 1/κ and the required phase ψ. For bistable subjects, the coefficients *a*_1_ and *a*_2_ are finite, all others vanish. During learning of the ψ = 90° pattern, the coefficient *a*_4_ and the parameter *c* increase from zero to finite values and at the same time the width of the distributions decreases. For simplicity, we assume that during the learning phase the coefficients and parameters are proportional to a single control parameter λ (Equations 5):

(5) $$a_{1}=1,\;a_{2}=3,\;a_{4}=0.6\;\lambda ,\;c=0.5\lambda ,\;\kappa =1.75\lambda$$

**Figure 4:**
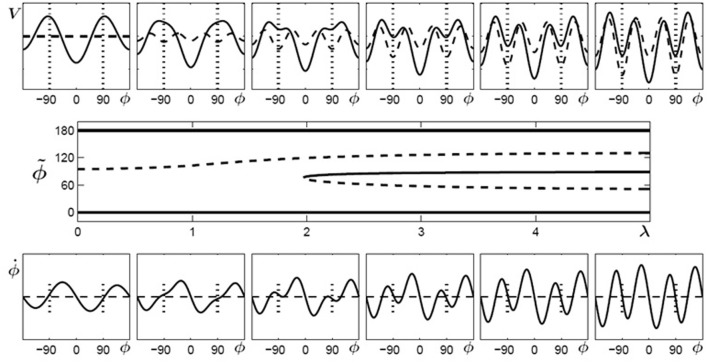
**Learning 90° for initially bistable participants**. **Top**: potential landscape, *V* solid, *V*_1_(λ) dashed; **bottom**: phase space portrait; **middle**: bifurcation diagram. With increasing λ, the unstable fixed point around 90° (dashed line) shifts towards anti-phase and near λ = 2 a saddle-node bifurcation occurs, leading to an additional unstable fixed point (dashed) and an attractor (solid) close to the required phase of ψ = 90° (only the upper half of the bifurcation diagram is shown since the part for negative values of ϕ is symmetric).

In Figure 4 (top) the potentials (solid curves) for parameter values of λ = 0… 5 are shown for initially bistable participants. The dashed curves indicate the contributions which change during the learning phase, i.e., the potential has two parts (Equation 6):

(6) $$V=\underset{\text{original skeleton}}{\underset{︸}{-a_{1}\cos\;\phi -a_{2}\cos\;2\phi }}-\underset{V_{1}\left(\lambda \right)}{\underset{︸}{a_{4}\cos\;4\phi -\frac{c}{2\pi I_{0}\left(\kappa \right)}\left\{e^{\kappa \cos\left(\phi -\psi \right)}+e^{\kappa \cos\left(\phi +\psi \right)}\right\}}}$$

where the first part represents the original skeleton prior to learning and the second part *V*_1_(λ) depends on the control parameter λ, essentially a temporal measure of how long learning takes place. In general, λ does not have to increase linearly with time but may saturate at a certain value. In Figure 4 (bottom), phase space portraits corresponding to the potential functions are shown. In these plots, an intersection between the curves and $\dot{\phi }=0$ (dashed) with a negative (positive) slope represent an attractor (repellor) in the system. The plot in Figure 4 (middle) shows a bifurcation diagram, the fixed points for the relative phase, $\tilde{\phi }$, against the control parameter λ, where solid (dashed) lines represent the locations of stable (unstable) fixed points. Around a value of λ ~ 2 the system undergoes a saddle-node bifurcation where an attractor-repellor pair appears, with the attractor representing the required relative phase 90°.

Similarly, for multistable participants, the coefficients *a*_1_, *a*_2_ and *a*_4_ are initially finite, all others vanish. During learning of the ψ = 135° pattern, the coefficient *a*_3_ and the parameter *c* increase from zero to finite values and at the same time the width of the distributions decreases. As described before, during learning we assume the coefficients and parameters to be proportional to a single control parameter λ (Equation 7):

(7) $$a_{1}\;=1,\;a_{2}=3,\;a_{4}=3,\;a_{3}=0.6\lambda ,\;c=0.9\lambda ,\;\kappa =1.75\lambda$$

**Figure 5:**
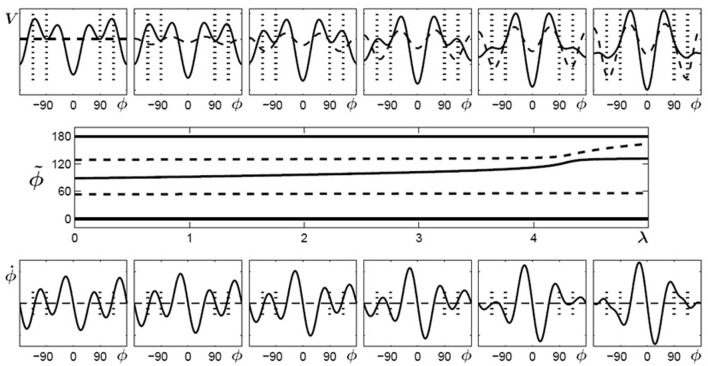
**Learning 135° for initially multistable participants**. **Top**: potential landscape, *V* solid, *V*_1_(λ) dashed; **bottom**: phase space portrait; **middle**: bifurcation diagram. With increasing λ, the stable fixed point around 90° (solid line) drifts towards the repellor (dashed) eventually leading to an attractor close to the required phase of ψ = 135° (only the upper half of the bifurcation diagram is shown because the part for negative values of ϕ is symmetric).

Using the same conventions as in Figure 4, Figure 5 displays potential landscapes, phase space portraits and corresponding bifurcation diagram for initially multistable participants. In this case, the number of attractors does not change, but the von Mises functions in combination with the cos3Φ term lead to a smooth shift of the stable fixed point around 90° toward the required phase of 135°.

**Figure 6:**
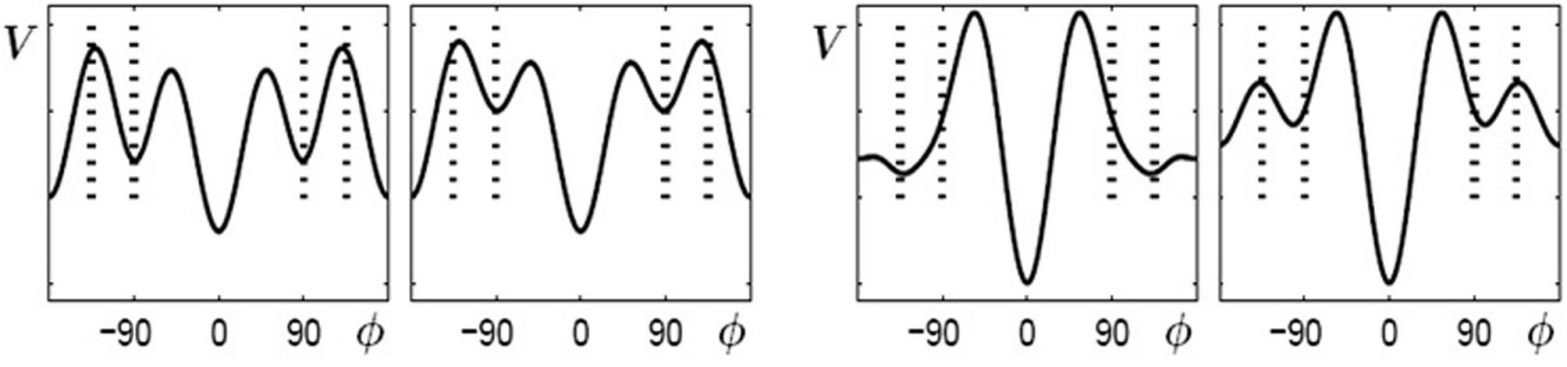
**Reorganization of potential landscapes when learning (or practice) has not taken place for some time**. **Left part**: bistable participants, where the minima at ±90° that were created during learning (left) persist (right). **Right part**: multistable participants where the minima located at ±135° after learning (left) shift back close to their initial values at ±90°.

When learning (or practice) no longer takes place the potential landscape reorganizes again but, as experiments show, not necessarily back to its initial shape. As described above, initially bistable participants can retain and perform the 90° pattern once it has been learned, whereas for initially multistable participants the stable coordination pattern learned at 135° shifts back towards 90° when learning stops. In the model these findings are captured by the skeleton (coefficients *a*_n_) and the change induced by the von Mises distributions acting on different time scales (Figure 6). The changes in the skeleton are long-lived, whereas the amplitude of the von Mises functions, given by the parameter c, decays relatively fast when learning has ceased. Obviously, the changes in the skeleton and the von Mises part cannot be described by the same parameter λ anymore, as the coefficients *a*_3_ and *a*_4_ remain at the value assumed at the end of the learning phase, whereas the contribution of the von Mises functions disappears on a short time scale, that is, the parameter c decays back to zero. This scenario is shown in Figure 6 for bistable (left part) and multistable (right part) participants, respectively. In both parts, the left plot corresponds to the end of learning (λ = 5) and the right plot to a later time where no learning or practice has occurred for a while with *c* = 0 but changes in the skeleton remain.

## Discussion

The present paper combines a program of empirical research and theoretical modeling that aims to reveal general principles and mechanisms of skill learning from an unorthodox perspective, namely, one that explicitly takes into account differences in individual behavioral predispositions that are present prior to learning. A central issue is to identify the nature of the changes due to learning and the factors that govern them. In conventional approaches, inspired mainly by machine learning concepts, learning is viewed as an error detection and correction process: any pre-existing tendencies that the individual learner brings into the learning environment about the task to be performed are largely ignored. Here, notwithstanding, experiment and theory point to the significance of individual dynamical properties in the learning process, namely, (behavioral) stability. Using bimanual coordination as a window, our experimental work shows that learning occurs indeed on the background of a pre-existing repertoire: the learner's initial intrinsic dynamics, whether it be bistable or multistable, determines the route that learning follows and the changes in behavior that occur. Learners possessing an initially bistable repertoire proved to follow a bifurcation route to learning, whereas those possessing an initially multistable repertoire follow a shift route, irrespective of the specific task to be learnt. The bifurcation route involves a larger gain in stability of the learned behavior and at higher attentional cost than the shift route, although similar levels of stability and accuracy may be reached when practice is over. Thus, beyond mere changes in behavior observed as improvements in performance, learning involves modifications at a deeper level, that of the individual's pre-existing (intrinsic) dynamics. The behavioral repertoire is altered in terms of the number of attractors (viz. spontaneously stable or preferred behavioral patterns) when learners take the route by bifurcation, whereas there is a gradual, transient displacement of an extant stable state toward the task requirements following the shift-route. These findings yield substantial support to growing evidence that, contrary to classic assumptions, there is no single pathway for behavioral change with learning (Ko et al., 2003; Jacobs, 2009; Stephen et al., 2010). For instance, using a perceptual learning paradigm, Jacobs et al. (2000), Michaels and Isenhower (2010) showed that from the plethora of perceptual variables available, different observers pick up different variables to guide perceptual learning. During training itself, learners change the variables they use in various ways. When the initial state of observers and the usefulness of perceptual variables are taken into account, learning can be understood as a predictable path through a perceptual space (Jacobs and Michaels, 2007). Here again, individual predispositions open a window into general rules for behavioral change.

Learning has long been associated with higher cognitive functions such as memory and attention. However, memory and attention have been studied separately and are, to date, associated with learning in no explicit fashion. Here, these cognitive functions may be coherently put back into relation by virtue of the fundamental dynamical notion of stability. Our findings indicate that the key argument is whether there is gain in stability with learning or not. In the case of the bifurcation route only is there any gain in stability, accompanied with a drop in attentional cost and the persistence of learned patterns in memory. In contrast, with the shift route, there is no gain in stability, no drop in attentional cost and no permanent storage in memory.

The foregoing conclusions are significantly backed up by theoretical modeling. In computer simulations, the creation of a novel attractor required by the learning task is captured by the Fourier development of the model skeleton, suggesting that the novel attractor arises through the very same bifurcation mechanism that governs change within the initially stable spontaneous patterns (viz. intrinsic dynamics). Such a skeleton creates the possibility of generating a series of maximally distant, symmetrically distributed attractors, each characterized by lower and lower stability. These internal constraints on the location, order and relative stability of the created attractors affect the effect of the von Mises distributions that formalize the shift route to learning. A key lesson is that learning emerges from a generic, internal rule (i.e., bifurcation) pertaining to the skeleton that is finely tuned as a function of its initial state, particular to each individual. Thus, for the first time to our knowledge, general and individual features of learning, as well as the learner's spontaneous and acquired capabilities are reconciled within a single model.

This model also reconciles two, apparently opposite descriptions of how learning is supposed to progress: learning by a gradual accumulation of tiny barely discernible steps is captured here by the shift route, whereas learning by abrupt changes, reminiscent of learning by insight (Kohler, 1925) corresponds to the bifurcation route. Continuous improvement points to the commonality of processes governing behavioral change of all living forms, whereas abrupt changes focus attention on individual specificities allowing qualitative transformations.

The specificity of the bifurcation route is to generate qualitative change in an initially bistable behavioral repertoire by adding new stable patterns. Bifurcation may thus be conceived as a dynamical mechanism for novelty. The selection of novel coordination patterns stabilized at specific values of relative phase is an instance of selection via instability (Kelso, 2000) and may be seen as a primordial mechanism without which further adaptive change may not be possible. Why is it so? By enriching the behavioral repertoire at maximally distant values of relative phase, the bifurcation mechanism establishes the best conditions for the shift mechanism to operate. When environmentally required and preexisting patterns are close enough, their influences cooperate and learning by shift is favored. When the required and preexisting patterns are far apart, they enter into competition with each other, which leads to instabilities, hence to novel pattern creation. A key lesson here is that self-organizing principles (viz. instabilities, bifurcations, fluctuations, effects of initial, and boundary conditions, etc.) define how the system interacts with environmental and task requirements. The more one knows about the individual learner's intrinsic dynamics, the more one can discover about the learning process. At any rate, the individual repertoire should never be considered a random or undifferentiated state—the very idea of a “blank slate”—that can be molded arbitrarily by the learning process (Kelso, 1995; Pinker, 2002).

The two concepts of initial repertoire (viz. intrinsic dynamics) and of bifurcation, central to the approach developed in the present paper, may be quite revealing for the field of behavioral neuroscience. First, our rendition of learning in terms of changes occurring at the level of the intrinsic dynamics finds an echo in the reorganization of neural networks (Wenderoth et al., 2008) and brain connectivity (Heitger et al., 2012) that follows the learning of a 90° pattern on top of the preexisting 0° and 180° coordination modes (Jantzen et al., 2002; see also Banerjee et al., 2012). Second, the process of bifurcation, at the heart, as we have seen, of learning, manifests itself clearly at the level of brain activity. Enhancement of fluctuations announcing the bifurcation phenomenon (see Introduction) has been confirmed in MEG and EEG recordings of the human brain (Kelso et al., 1992; Mayville et al., 2002). Moreover, a study by Meyer-Lindenberg et al. (2002) demonstrated that a transition between the two bimanual patterns constitutive of bistable coordination dynamics can be elicited by transient transcranial magnetic stimulation (TMS) of relevant brain regions such as premotor and supplementary motor cortices. In keeping with the stability~instability principle of coordination dynamics (Kelso and Engstrøm, 2006), such TMS perturbations caused a behavioral transition from the less stable anti-phase pattern to the more stable in-phase pattern, but not vice-versa.

That the stability of coordination pattern is a governing factor in how the brain works is further illustrated in a recent fMRI study by Jantzen and colleagues (2009). In a sensorimotor coordination paradigm in which participants had to coordinate in-phase (synchronize) or antiphase (syncopate) with an external auditory pacing stimulus, a clear dissociation was found between neural regions that are activated when a control parameter changes and neural regions connected to the stability of the coordination pattern. A key result was that the activation of cortical regions supporting coordination (e.g., left and right ventral Premotor Cortex, Insula, pre SMA, and cerebellum) scaled directly with the stability of the coordination pattern. As the anti-phase pattern became increasingly less stable and more variable, so too did activation of these areas. Thus, these areas of the brain, which form a functional circuit, have to work harder to maintain coordination in the face of increasing environmental demands. Task difficulty, in other words, often described in terms of information processing load, can also be captured by the dynamic measure of stability and is directly and lawfully related to the amount of energy used by the brain. Importantly, for identical control parameter values the same brain regions do not change their activation at all for the more stable (less variable) in-phase pattern. Given the constraints, a path is chosen that tends to favor the most stable state, here the potential minimum of an informational quantity, the relative phase.

The Jantzen et al. paper also shows how multistability is realized by the same cortical circuitry which itself is highly sensitive to the (in) stability of behavior. Together with the Meyer-Lindenberg et al., study this work illustrates the power of coordination dynamics to predict the dynamical repertoire of the brain. Though only beginning to be appreciated in the brain sciences (Plenz and Thiagarian, 2007; Chialvo, 2010; Kelso, 2010), dynamic stability and instability appear to be major determinants of the recruitment and dissolution of brain networks, providing flexibility in response to control parameter changes. One may wonder why the brain implements neural processes that eventually lead to short-lived behaviors, brought about by the shift route on the background of multistable dynamics. Multistability confers a tremendous selective advantage to the brain and to nervous systems in general: it means that the brain has multiple patterns at its disposal and can switch among them to meet environmental or internal demands. Shifting attractors among coexisting functional states on exposure to a new set of conditions is potentially more efficient than having to create states de novo. This hypothesis can be examined further by studying how different combinations of sound, touch, vision and movement come together and spontaneously split apart in time as parameters are varied (e.g., Kelso et al., 2001; Lagarde and Kelso, 2006).

A key notion in our approach to learning is that of intrinsic dynamics. It is a truism in psychology—hearkening back at least to Watson (1909)—that we do not come into the world as a *tabula rasa* but with a repertoire of existing dispositions, capacities and basic abilities. In fact, it is only in the last 20 years or so that new concepts, strategies and tools of coordination dynamics have provided a quantitative means to probe the current state of the learner's repertoire and to follow its evolution as learning proceeds. The challenge has been to find a paradigm and a methodology that afford inroads into dynamic principles and mechanisms of learning. Depending on the individual's initial repertoire adaptive changes due to learning are governed by a shift mechanism or a bifurcation mechanism. Unlike the former, bifurcation is a mechanism pertaining to attention, characterized by an initial loss of stability and resulting in long-lived qualitative changes of the behavioral repertoire. In this respect, numerous works stemming from a Bayesian approach have recently focused on the impact of prior skills on learning. The Bayesian framework (see Mamassian et al., 2002; Shadmehr et al., 2010, for an introduction) suggests that on the basis of past experience, the brain lawfully generates a set of predictions, called priors, about the environmental states. Then, these predictions are combined with sensory likelihood, computed from the sensory input, to form the best estimate of the real state of the world. There are two key differences between Bayesian proposals and our dynamic approach to learning.

First, coordination dynamics insists on the interplay between self-organizing processes and environmental or task requirements, whereas a Bayesian approach pictures learning as a process built up exclusively on experience. Thus, our experiments explore the effect of spontaneously arising, inter-individual differences on learning, evidenced in scanning probes before learning, whereas a Bayesian approach establishes prior skills during a pre-learning session (Körding and Wolpert, 2004; Huang and Shadmehr, 2009). In Bayesian framework, learners, viewed as blank slates, are first trained for 1000 trials and only then, the effect of the just-trained skills, called “priors,” is assessed on (Körding and Wolpert, 2004). A second difference pertains to the role attributed to variability. In a Bayesian framework, the actual variability is seen as an error-inducing source of uncertainty, which must be corrected during the planning of movements (Trommershäuser et al., 2008). It is thus interpreted as a non-functional noise limiting the information conveyed by neural commands (Fitts, 1954; but see also Kelso, 1992; Slifkin and Newell, 1998, 2000; Deutsch and Newell, 2006 for an appraisal). In a dynamic framework, variability is a hallmark of loss of stability heralding bifurcation. Thus, variability is always present, testing whether a given pattern is stable and allowing the system to switch to new, more stable patterns.

Our perspective also comes to grips with classic error-centered models of learning, in which error reduction drives learning mechanisms (Adams, 1971; Schmidt, 1975; Körding and Wolpert, 2004; Berniker and Kording, 2008; Friston, 2010). Our findings indicate that the evolution in accuracy is predictive of neither the changes in the attentional cost with learning nor performance at recall. Contrary to intuition, the learning process does not operate by choosing or eliciting more and more accurate behaviors. Indeed, despite years of extensive training, golfers may not progress beyond the amateur level (Ericsson, 2003). Despite many learning trials, the reference necessary to error computation may be impossible to perceive (Withagen and van Wermeskerken, 2009) and learners can fail in the perceptual search for relevant features (Jacobs, 2009). Moreover, during the process of skill learning, children often regress to earlier, less efficient movements (Corbetta and Bojczyk, 2002; Langendorfer and Roberton, 2002). Our contention, based on both data and theoretical modeling, is that learning is always geared to maximize stability of a behavioral pattern^3^.

Although the theoretical model proposed here was developed to account for elementary forms of sensorimotor learning, it may also be quite insightful regarding changes in behavior that occur on other time scales, in particular development (Thelen et al., 1987; Sporns and Edelman, 1993; Thelen and Smith, 1994), perceptual categorization (Tuller et al., 2008), and biological evolution (Gould and Eldredge, 1993). For instance, recent work on the acquisition of handwriting (Danna et al., 2012) suggests that changes in the way that children and adults produce a written trace may be ascribed to a bifurcation process in the underlying dynamics in which an existing attractor located at 90° is replaced by two new ones located at 60° and 120°. Our perspective is also in line with recent thinking on the origin and evolution of life. Contrary to most models of biological evolution (e.g., Kauffman, 1993), Root-Bernstein and Dillon (1997) argue that life did not develop from random interactions of molecules inside the original soup of chemical components. Chemical components have been able to enter in interaction and exercise an action on each other only if the spontaneously appearing form allows them to establish such an intimate relationship. When the form is complementary (Graben beim and Atmanspacher, 2006), such as a lock and a key, interacting components create assemblies for non-trivial durations, resisting perturbation and supporting functional exchanges. Then, they co-evolved on a longer time scale, bringing about more complex and sophisticated assemblies. Scrutinizing the initial state of system is therefore a primordial step toward understanding all kinds of change at many different levels of description and times scales for various functions.

### Conflict of interest statement

The authors declare that the research was conducted in the absence of any commercial or financial relationships that could be construed as a potential conflict of interest.

## Footnotes

^1^Imagine if an individual's preexisting repertoire is not known in advance (the usual case) and the learner “learns” the task immediately. A possible reason, which cannot be discounted unless the preexisting repertoire is known, is that the arbitrarily chosen task is already part of the repertoire.

^2^As in indication, the Psychline database (2012) indicates that among 8511 articles dealing with motor learning, only 4.7% also mention both memory and attention.

^3^While intrinsic dynamics and maximizing stability appear to be guiding principles in learning the current coordination task, it is reasonable to inquire whether these principles might also apply more generally to learning complex motor and perceptual skills. There are reasons to conclude that they do. It is always a question whether the scientist's reduction to a laboratory frame of reference captures the essence of the real thing (Kelso, 1995, p.53ff.; see also Krakauer and Mazzoni, 2011). A plus about bimanual skill learning is that it retains certain essential features of all skills such as adaptation, stability, flexibility, switching and so forth—all of which are measurable—while pruning away many of the real-life complications typical of naturally occurring behaviors. The task requires each finger or limb to get to the right place at the right time in order to meet environmental requirements (Kelso et al., 1998) and the limbs themselves have to be precisely coupled so as to produce a particular order in time. It is not by chance that bimanual coordination has been referred to as a prototype of complex skill (Swinnen, 2002). The various conceptual and methodological aspects associated with bimanual coordination dynamics have been widely adopted to understand other skills such as speech production (e.g., Tuller and Kelso, 1990; Port, 2003; Simko and Cummins, 2010) and handwriting (*op.cit*., Danna et al., 2012). With respect to intrinsic dynamics, it is striking to note that despite the variety of environmental demands in natural settings, inphase and antiphase arise as the main preferred patterns (Howard et al., 2009). Also, choreographers teaching improvisation techniques underscore that dancers are challenged by the demand of producing original motor patterns: they tend to be ‘trapped’ in familiar ones (Hagendoorn, 2003). It appears that were there no instrinsically stable patterns to buffer short-lived environmental demands, the system would be subject to continuous perturbations, without ever being able to settle down on any pattern.
